# Spontaneous Intestinal Perforation in Neonates Involving the Cecum: A Case Report

**DOI:** 10.7759/cureus.37322

**Published:** 2023-04-09

**Authors:** Raju K Shinde, Rahul Rajendran

**Affiliations:** 1 Department of General Surgery, Jawaharlal Nehru Medical College, Datta Meghe Institute of Medical Sciences, Wardha, IND; 2 Department of Surgery, Jawaharlal Nehru Medical College, Datta Meghe Institute of Medical Sciences, Wardha, IND

**Keywords:** hirschsprung disease, necrotizing enterocolitis, neonate, idiopathic perforation, cecum

## Abstract

Neonatal spontaneous colonic perforation in term neonates is a rare phenomenon, with caecal perforation being seldom reported. Therefore, this case report presents a rare case of spontaneous caecal perforation in a term neonate who presented with vomiting and abdominal distension on day two of life. On exploration, a single large full‑thickness cecum perforation was noted. Histopathologic samples were negative for necrotizing enterocolitis and Hirschsprung’s disease. Clinical awareness of this rare entity could help in preventing delays in imaging and prompt surgical management.

## Introduction

A gastrointestinal perforation is a neonatal surgical emergency. Although small intestinal perforation is frequently seen in newborns, reports of colon perforation are rare. In preterm infants, colonic perforation is caused mostly due to necrotizing enterocolitis (NEC). It may be associated with Hirschsprung disease, small left colon syndrome, meconium plug syndrome, idiopathic perforations, anorectal malformations, stercoral perforations, and cystic fibrosis [1-3]. In term and near‑term infants, Hirschsprung disease is the leading cause, followed by NEC and spontaneous idiopathic perforation [1-4]. Bowel perforation that develops idiopathically otherwise known as spontaneous intestinal perforation has no known cause. As early as 1825, it was initially noted in newborns. In a term newborn, idiopathic cecum perforation is incredibly rare; hence, this case report presents a case of a term female neonate who suffered a single idiopathic cecum perforation.

## Case presentation

**Figure 1:**
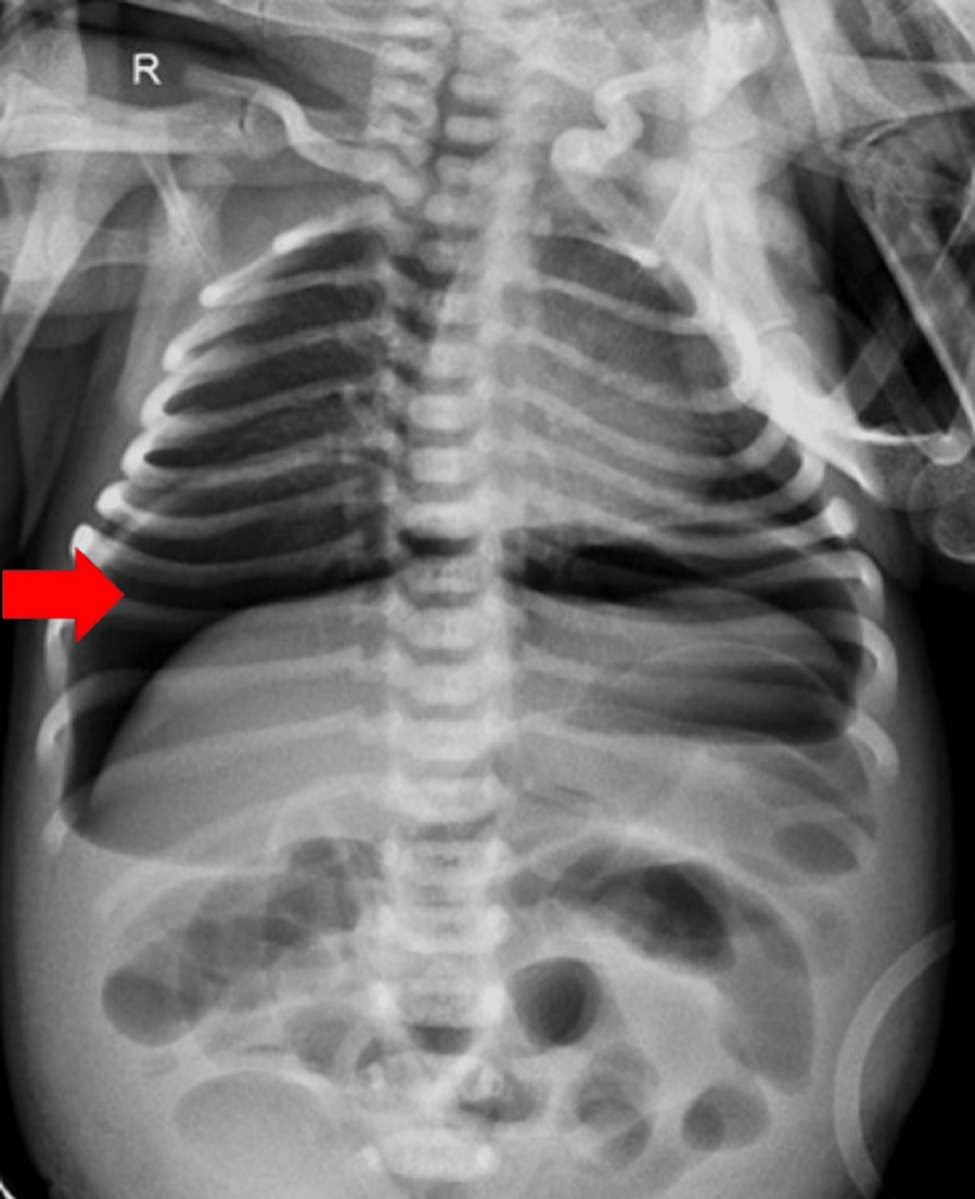
X-ray of the abdomen showing massive pneumoperitoneum illustrated by a red arrow

A two-day-old female with complaints of vomiting and distension since day one was referred to our hospital. She was a term baby weighing 2.6 kg born to a 25-year-old primigravida by cesarean section. The baby cried soon after birth but had not passed meconium in the first 24 hours. Since the mother had decreased secretions, the baby was fed water mixed with sugar. The last few months of pregnancy went without incident. There was no evidence of any family history of the condition. Upon clinical examination, she displayed decreased activity, and her heart rate was 180 beats per minute with dehydration. Her abdomen was firm but not red, and she did not have any bowel sounds. A rectal examination indicated no clutching and an empty rectum. A significant pneumoperitoneum was visible on both sides of the abdomen on a plain X-ray taken with the patient's abdomen upright (Figure 1).

**Figure 2:**
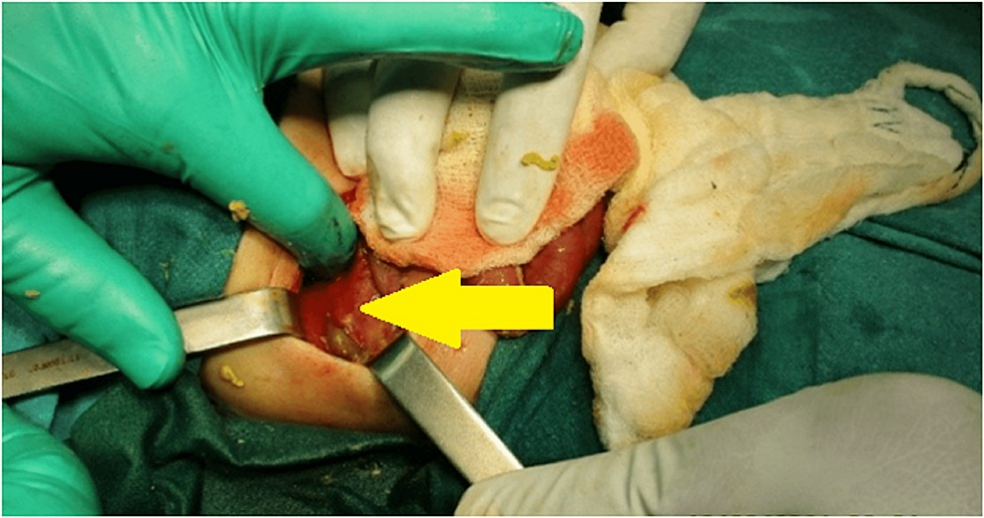
Intraoperative image showing single full-thickness cecum perforation illustrated by a yellow arrow

**Figure 3:**
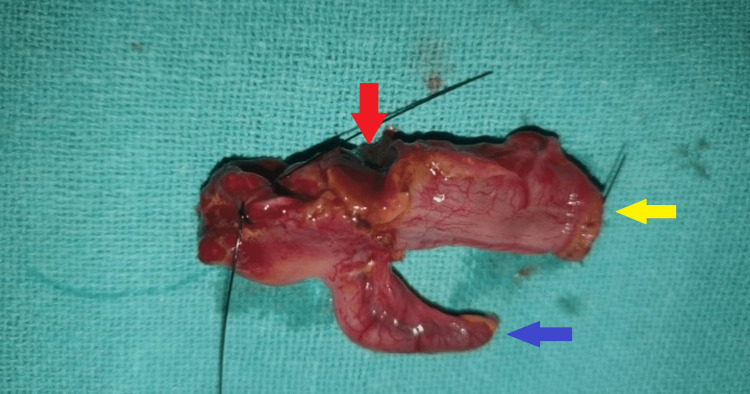
Resected specimen of terminal ileum illustrated by a yellow arrow, the appendix by a blue arrow, and the proximal cecum with a single full-thickness perforation by a red arrow

An infant feeding tube was inserted once peritonitis due to perforation was diagnosed. The administration of intravenous fluids and antibiotics was initiated. Exploratory laparotomy was done by the right upper transverse supraumbilical incision, and the peritoneum showed fecal contamination. One large full-thickness perforation (0.5 × 0.5 cm) was identified in the cecum, and the rest of the bowel was found to be dilated (Figure 2 and Figure 3).

Resection of the terminal ileum, appendix, and part of caecum with double barrel ileostomy was done. Rectal biopsies were taken. However, the biopsy report did not identify any particular pathological diagnosis. The post-operative period was uneventful; the stoma became functional on postoperative day one and oral feeds were started on postoperative day five. The patient responded well to the treatment regimen.

## Discussion

Hirschsprung disease is perhaps the only condition where a routine correlation to a cecum perforation has been made in case studies [1,2,4]. The incidence of 3%-4% is, however, largely seen in long‑segment Hirschsprung disease. Another etiology that is closely related is NEC. However, NEC is generally found in preterm babies. If perforation occurs, the ileum and jejunum are typically involved in preterm newborns. The most significant etiological elements in the pathophysiology of NEC have been found to be intestinal mucosal damage resulting from poor oxygen circulation conditions and associated bacterial overload [2,3,5]. Idiopathic perforation of the cecum has almost always been reported in isolated case reports as a rare entity.

Most of these cases are diagnosed after the possibilities of a probable etiology have been carefully excluded. Resch et al. suggested that the combination of episodes of hypoxia, resulting in ischemic injury to the gut, together with the immaturity of the intestine could result in perforation in these infants [6]. Theories for spontaneous colonic perforation in neonates include bacterial invasion of the intestinal wall and endotoxins-induced Schwartzman reaction. Gastric and colonic perforation should be the differential diagnosis for a massive pneumoperitoneum in a neonate. A classical "saddle sign" will be visualized on an upright abdominal film. Neonates with spontaneous intestinal perforations can be treated by drain alone, drain and laparotomy later, or primary laparotomy [7-9].

An optimal management plan depends on the site of perforation, degree of contamination, and hemodynamic stability of the neonate. In a hemodynamically stable patient with a well-localized and single perforation primary closure or resection and anastomosis can be performed. In cecum perforations, unless Hirschsprung disease is ruled out, a primary repair becomes impossible, and a stoma is usually done. Biopsies are recommended in all cases [10]. Availing frozen sections is of great advantage in immediate decision making, but is a limitation as it is not available in most centers. As the ileocecal valve was not salvageable, an ileostomy had to be performed. The morbidity of these patients remains high, with the stoma output needing extensive resuscitation and intensive care unit care. In spite of efforts, the mortality rate in most studies is close to 50% [1,2,6]. The patient had a spectacular outcome and is growing well after the completion of surgical treatment. The final diagnosis considered was idiopathic perforation of the cecum as there was no clinical, radiological, or histopathological evidence of diseases like necrotizing enterocolitis, Hirschsprung’s disease, or meconium ileus and related diseases.

## Conclusions

Spontaneous perforation of the cecum in a neonate without a demonstrable or known cause is sufficiently rare. It is extremely important to perform a thorough history and histopathological evaluation in all cases. The management varies depending on the cause. Even with the best care, babies with colonic perforation tend to have a poor prognosis.
